# Risk and Protective Factors of Depression in Family and School Domains for Chinese Early Adolescents: An Association Rule Mining Approach

**DOI:** 10.3390/bs13110893

**Published:** 2023-10-29

**Authors:** Chen Wang, Ting Zhou, Lin Fu, Dong Xie, Huiying Qi, Zheng Huang

**Affiliations:** 1 Department of Health Informatics and Management, School of Health Humanities, Peking University, Beijing 100191, China; wangchenparis@bjmu.edu.cn; 2 Department of Medical Psychology, School of Health Humanities, Peking University, Beijing 100191, China; zhouting.92@bjmu.edu.cn; 3 Faculty of Humanities and Social Sciences, Beijing University of Technology, Beijing 100124, China; linfu@bjut.edu.cn; 4 School of Basic Medical Sciences, Peking University, Beijing 100191, China; 1910305322@pku.edu.cn; 5 Department of Psychology, University of Chinese Academy of Sciences, Beijing 100101, China; 6 Institute of Psychology, Chinese Academy of Sciences, Beijing 100101, China

**Keywords:** adolescent depression, age group, association rule mining, gender, left-behind status, protective factors, risk factors

## Abstract

Depression is one of the most common psychological problems in adolescence. Familial and school-related factors are closely related to adolescents’ depression, but their combined effects need further examination. The purpose of this study was to explore the combined effects of risk/protective factors of depression in family and school domains using a sample of Chinese adolescents differing in gender, age group and left-behind status. A total of 2455 Chinese students in primary and secondary school participated in the cross-sectional survey and reported multiple risk/protective factors in family and school environments and depressive symptoms. Association rule mining, a machine learning method, was used in the data analyses to identify the correlation between risk/protective factor combinations and depression. We found that (1) Family cohesion, family conflict, peer support, and teacher support emerged as the strongest factors associated with adolescent depression; (2) The combination of these aforementioned factors further strengthened their association with depression; (3) Female gender, middle school students, and family socioeconomic disadvantages attenuated the protective effects of positive relational factors while exacerbating the deleterious effects of negative relational factors; (4) For individuals at risk, lack of mental health education resources at school intensified the negative impact; (5) The risk and protective factors of depression varied according to gender, age stage and left-behind status. In conclusion, the findings shed light on the identification of high-risk adolescents for depression and underscore the importance of tailored programs targeting specific subgroups based on gender, age, or left-behind status.

## 1. Introduction

Related to pubertal development and increasing social demands, the incidence of depression in adolescence is higher than that in childhood and has increased significantly in recent years [1]. The prevalence of depression disorders in Chinese children and adolescents aged 6–16 years is 3.0% [2], and 14.8% of adolescents experience high depressive symptoms [3]. Given that depressive symptoms affect adolescents’ daily function and academic achievement, and have a long-term negative impact on adolescents’ mental health and social adjustment [1], it is important to identify risk and protective factors for depression in Chinese adolescents and develop effective preventive and intervention programs.

According to the social-ecological system theory [4], family and school play pivotal roles in influencing the social development of adolescents. Revealing the risk and protective factors within the family and school environments and comprehending the interaction mechanisms among different factors can facilitate the identification of students at high risk of mental health, enabling focused attention and targeted intervention. Moreover, it facilitates effective coordination of resources between families and schools in order to create favorable environments in both settings and to prevent and control mental health problems such as adolescent depression.

Previous studies conducted in China and Western countries have identified numerous family factors associated with depressive symptoms in children and adolescents, including low family socioeconomic status [5], negative events in early life [6], parents’ depression problems [7], parent-child communication problems [8], harsh parenting [9] and a negative family emotional climate [10]. In a meta-analysis conducted by [11] on Chinese middle school students’ depression-related factors, it was found that among these family factors, parent-child communication problems, low family function and cohesion were particularly strongly correlated with depression at a medium to high level [11]. Conversely, positive family function can improve individual psychological resilience and have a positive predictive effect on mental health outcomes [12].

Most Chinese adolescents spend their teenage years immersed in the educational system. Research has consistently demonstrated that experiences of social isolation and bullying in the school setting are important predictors of depression in children and adolescents [13,14]. Furthermore, academic pressure and poor academic performance are also significantly correlated with adolescent depression [15]. However, fostering a positive school atmosphere, and providing support from teachers and peers can effectively promote students’ positive development [16,17]. Additionally, the availability of psychological education resources within schools plays a protective role in enhancing students’ mental health [18].

Risk and protective factors in the family and school environments concurrently influence adolescents’ mental health. Previous research has indicated that students with a familial history of depression can benefit from positive relationships with another parent and school connectedness [19,20]. In this case, school protective factors have the potential to mitigate the negative impact of family risk factors. However, there might be other interaction patterns between school and family factors. For example, previous research has indicated that students facing higher family risks are less likely to benefit from school resources [21]. Therefore, gaining a comprehensive understanding of the risk and protective factors as well as their interactions, can facilitate the development of targeted mental health education programs aimed at preventing and managing adolescent depression.

It should be noted that the risk and protective factors for depression in adolescents may differ by gender. The depression prevalence in females is higher compared to males in any age group from adolescence [22]. Because of gender inequality in most societies, females experience greater stress in daily life and social pressures to conform to gender roles increase when children move through puberty [23]. In addition, females have a greater tendency to be concerned with relationships with others and others’ opinions of themselves; thus, they are at higher risk for depression when confronting conflicts in relationships [24]. There is also evidence that depression in adolescent boys is more strongly correlated with harsh parenting behaviors [11]. Thus, it is necessary to identify risk and protective factors for depression separately for adolescent girls and boys.

Regarding the age difference, it has been found that depression prevalence usually increases during the period of adolescence and reaches its peak in mid to late adolescence. Nevertheless, it has been found that students in primary school have exhibited a relatively high prevalence of depressive symptoms [25]. The quantity and quality of risk factors for depression might vary at different stages of pubertal development and the socialization process. Therefore, this study also explored the risk and protective factors of depression for primary school students (11–12 years old) and middle school students (13–15 years old) separately.

In addition to gender and age, this study also attempted to compare the family and school risk and protective factors of depression in left-behind and non-left-behind students. Due to the imbalance of economic development, young adults in underdeveloped areas of China go to work in developed areas and leave their children in their hometowns. As revealed by a number of studies, left-behind children and adolescents are at higher risk of depression compared to their non-left-behind counterparts [26]. From the perspective of improving the mental health of left-behind adolescents, it is worth focusing on whether school resources can play a compensatory role for disadvantages in family resources, and further, which school factors effectively promote the mental health of left-behind adolescents.

In summary, although a large number of family and school factors have been found to be associated with depressive symptoms in adolescents, there remains a research gap in this field. Firstly, most previous studies have primarily focused on examining the individual or limited factors related to depression, neglecting comprehensive exploration of the joint effects of multiple familial and school-related factors. Association rule mining has not been used to address this issue to our knowledge. Secondly, given the difference in depression prevalence by gender, age and left-behind status, it is imperative to examine the risk and protective factors for depression among adolescents with different gender, age, and left-behind status to facilitate targeted intervention.

Aiming at deepening the understanding of the risk and protective factors of depression in young people and their joint effects, a cross-sectional investigation was conducted with primary school students and middle school students as participants. Association rule mining, a data mining technique used to discover relationships or patterns between item sets or object sets in large datasets, was used to explore factor combinations associated with depressive symptoms for adolescents with different gender, age and left-behind status. By using association rule methods for mutual relationship analysis, it is easier and more effective to obtain relevant rules between multiple variables and provide valuable insights, thereby improving the decision-making process. The specific research questions were as follows: (1) exploring and comparing risk factor combinations associated with depression and protective factor combinations related to nondepression for adolescent girls and boys; (2) exploring and comparing risk factor combinations associated with depression and protective factor combinations related to nondepression for students in primary schools and middle schools; (3) exploring and comparing risk factor combinations associated with depression and protective factor combinations related to nondepression for left-behind and non-left-behind students.

This study extends previous research by revealing the combined effects of multiple protective/risk factors in family and school domains and specifying protective/risk factors for subgroups of adolescents differing in age, gender and left-behind status with a novel machine learning approach. This study also contributes to the field of school psychoeducation by providing evidence for the identification of high-risk students and the development of targeted interventions.

## 2. Method

### 2.1. Participants and Procedure

This study received approval from the Ethics Committee of our institute. Students were recruited in two counties located in southern and northern China and the two counties had a medium GDP in China. The cluster sampling method was employed and 38 primary schools and 11 middle schools were involved. Consent was obtained from students and their parents, ensuring ethical compliance. The paper-based questionnaires were administered to students during class sessions. A total of 2800 students were recruited, and 2445 of them provided completed data for analyses. Students were acknowledged for their participation and received a small token of appreciation. Among the participants, 1292 (52.8%) were girls and 1153 (47.2%) were boys. Among the primary school participants (*n* = 1164), there were 590 (24.1%) fifth graders and 574 (23.5%) sixth graders. The middle school sample (*n* = 1281) comprised 414 (16.9%) seventh-grade students, 418 (17.1%) eighth-grade students and 449 (18.4%) ninth-grade students. Additionally, there were 870 (35.6%) left-behind adolescents in the entire sample.

Data were entered using Epidata 2.1 software, with double-entry verification implemented to ensure data accuracy.

### 2.2. Measures

#### 2.2.1. Demographic Questionnaire

Demographic information including gender, grade, and ethnicity was collected. In addition, data on family structure, parents’ education attainment, left-behind status of children, family economic status and academic ranking were also gathered. The description of the variables is listed in Table 1.

#### 2.2.2. Family Cohesion

The cohesion subscale in the Family Adaptation and Cohesion Evaluation Scales II-Family Version (FACES-II) [27] was utilized in the present study. There are 16 items on the scale measuring closeness among family members. An exemplar item is “Family members experience a strong sense of emotional closeness”. Ratings for these items were obtained using a 5-point Likert scale ranging from 1 (almost never) to 5 (almost always), with higher scores indicating greater levels of family cohesion. The Chinese version of FACES-II demonstrated satisfactory reliability and validity [28]. In the present study, Cronbach’s α was 0.82. As displayed in Table 1, participants were categorized as being at risk if their total score fell below the 25th percentile, while those scoring above the 75th percentile were considered to possess protective resources [29].

#### 2.2.3. Family Conflict

The 9-item conflict subscale of the Family Environment Scale [30] was used to measure family conflicts. An example item is “Family members often blame and criticize each other”. The items are rated on a dichotomous scale (no = 0, yes = 1). Total scores are calculated and with higher scores indicating higher levels of family conflict. The Chinese version of this scale shows good psychometric properties [28]. The Cronbach’s α was found to be 0.63 in the present study. Scores above the 75th percentile were categorized as indicating risk, while scores below the 25th percentile were categorized as protective [29].

#### 2.2.4. School Climate

Teacher support, peer support and autonomy support were measured using the school climate scale [31]. The scale consisted of seven items tapping teacher support (e.g., “teachers believe I can do well”), 13 items tapping peer support (e.g., “students care about one another” and five items tapping opportunities provided for autonomy in the school (e.g., “students are given the chance to help make decisions”). Participants rated these items on a 4-point Likert scale ranging from 1 (“never”) to 4 (“always”), with higher scores indicating greater levels of teacher support/ peer support /autonomy support. The Cronbach’s α values were 0.80, 0.81, and 0.76 for each subscale, respectively. As demonstrated in Table 1, scores below the 25th percentile in the three subscales were deemed to indicate a higher level of risk, while those above the 75th percentile were considered to possess protective resources [29].

#### 2.2.5. Mental Health Education Resources in Schools

Three questions were used to investigate the provision of mental health education in schools, specifically, “Does your school offer psychology courses?”, “Are there psychological counselors available in your school?” and “Does your school have a psychological consultation facility”. The criteria for risk and protection in terms of mental health education resources are displayed in Table 1.

#### 2.2.6. Adolescent Depression

The Depression Self-Rating Scale for Children (DSRSC, Ref. [32] was employed to assess depressive symptoms. The DSRSC is appropriate to assess depressive symptoms in children aged 8 to 16 years. This scale comprises 18 items that are rated on a 3-point scale (0 = never, 1 = sometimes, 2 = often), with higher scores indicating greater levels of depressive symptoms. The Chinese version of the DSRSC has demonstrated robust psychometric properties and a cutoff point of 15 has been proposed to differentiate adolescents at low and high risk of depression [33]. In this study, the internal consistency of the DSRSC was deemed acceptable (α = 0.84).

### 2.3. Data Analyses

In machine learning algorithms, the association rule is used to find frequent patterns, associations, correlations, or causal structures that exist between sets of items or objects in datasets [34]. By analyzing the correlation between multiple attributes in the data, the association rule discovers valuable rules. The basic meaning is as follows:

Let $I=\left\{i_{1},i_{2},\ldots ,i_{m}\right\}$ be an item set, where $i_{k}\left(k=1,2,\ldots ,p\right)$ represents an item, that is, a prediction variable. Let $D=\left\{d_{1},d_{2},\ldots ,d_{n}\right\}$ be a transaction set, where $d_{j}=\left\{i_{1},i_{2},\ldots ,i_{k}\right\}$ is a set containing k items, called a k-item set (e.g., {gender, depression} is a 2-item set), and $d_{j}\subseteq I$. The form of the association rule is X⇒Y, where X⊂I, Y⊂I and X∩Y=∅. X and Y are both item sets, and they are referred to as the left-hand side (LHS) and right-hand side (RHS) of the association rule, respectively.

The association rule evaluates the association strength through support, confidence and lift. The definitions and formulas of these evaluation parameters are shown in Table 2.

To meet certain requirements, it is necessary to specify the support and confidence thresholds. X⇒Y is considered valuable when $\sup\left(X\Rightarrow Y\right)$ and $\mathrm{conf}\left(X\Rightarrow Y\right)$ are greater than or equal to the set threshold value, respectively. These two values are called min_sup and min_conf, min_sup describes the minimum importance of association rules, and min_conf specifies the minimum reliability that association rules should meet.

However, the settings of min-sup and min_conf have a significant impact on the final result. The min_sup is too large, and plenty of potential rules may be deleted. In contrast, many redundant rules could be generated, making it difficult to study and discover association relationships. Therefore, taking the lift $\left(X\Rightarrow Y\right)>1$ as the basic requirement for effective strong association rules and based on the principle of not omitting important rules, min_sup and min_conf are set through multiple threshold setting experiments.

This study used the Apriori algorithm [35] of the association rules to identify risk factors related to depression and protective factors linked to nondepression by analyzing the relationship between predictive variables and outcomes. The implementation process was divided into two parts: (1) discover frequent item sets, defined as item sets with support greater than or equal to the given min_sup, and (2) generate association rules. Subsequently, effective strong association rules were obtained based on different support, confidence and lift parameters, and then the influencing factors behind the relevant results were analyzed.

## 3. Results

### 3.1. Risk Factors for Depression: The Association Rules by Gender, Age Group and Left-Behind Status

To identify the risk factors associated with depression, RHS is set as depression in order to establish association rules. By taking lift (X⇒Y) > 1 as the basic requirement, we compared the changes in association rules, and ultimately applied min_sup = 5% and min_conf = 70% for models by gender and age group, and min_sup = 5% and min_conf = 80% for the model by left-behind status. The combinations of risk factors that meet the above parameter conditions were identified as the significant association rules in this study. The generated association rules were arranged in descending order of confidence degree, as shown in Table 3, Table 4 and Table 5. In tables, support reflects the probability of both risk factors and depression occurring simultaneously, and confidence reflects the probability of depression occurring under the conditions of risk factors occurring. Lift reflects the correlation between risk factors and depression, with a value greater than 1, indicating a positive correlation between the two.

#### 3.1.1. Risk Factors of Depression by Gender

Table 3 displays a summary of the association rules by gender group. For males (Part A), 2 strong association rules were derived, both of which were 3-item sets. The only 3 risk factors among the 12 risk factors(shown in Table 1) used as LHS that are included in the association rules are low family cohesion, high family conflict and low teacher support. The low family cohesion-high family conflict combination has the highest correlation with depression (lift = 3.04).

For females (Part B), 28 strong association rules were derived, of which 1 was 5-item sets, 12 were 4-item sets and 15 were 3-item sets. The 10 risk factors among the 12 risk factors used as LHS were included in the association rules. In addition to the three risk factors that appear in males, they also included low peer support, absence of counselors or counseling rooms in the school, separation from parents, low parental education level, family economic strain and absence of psychological courses. The factor of middle school students also appeared in association rules. The low family cohesion-high family conflict combination, and the absence of counselors in the school combination had the highest correlation with depression (lift = 2.61).

Support of association rules by gender ranged from 5.03% to 9.91%, confidence ranged from 70.21% to 89.04%, and lift ranged from 2.06 to 3.04.

#### 3.1.2. Risk Factors of Depression by Age Group

The association rules by age group are illustrated in Table 4.

For primary school students (Part A), 1 strong association rule was derived, of which was 3-item sets. The only 2 risk factors among the 12 risk factors used as LHS that were included in the association rules were low family cohesion and low peer support. The low family cohesion-low peer support combination had the highest correlation with depression (lift = 2.54).

For middle school students (Part B), 30 strong association rules were derived, of which 1 was 5-item sets, 15 were 4-item sets and 14 were 3-item sets. The 11 risk factors among the 12 risk factors used as LHS were included in the association rules. In addition to the two risk factors that appear in primary school students, they also included high family conflict, low teacher support, low parental education level, absence of counselors or counseling room in the school, family economic strain, low autonomy support, separation from parents and absence of psychology courses. The factor of females also appeared in association rules. The high family conflict-low family cohesion, and low peer support combination had the highest correlation with depression (lift = 2.81).

Support of association rules by age group ranged from 5.15% to 9.99%, confidence ranged from 70.21% to 90.54%, and lift ranged from 2.18 to 2.81.

#### 3.1.3. Risk Factors of Depression by Left-Behind Status

Table 5 summarizes the association rules for risk factors of depression by left-behind status. For non-left-behind children (Part A), 1 strong association rule was derived, of which was 3-item sets. The only 2 risk factors among the 12 risk factors used as LHS that were included in the association rules were high family conflict and low family cohesion. The high family conflict-low family cohesion combination had the highest correlation with depression (lift = 2.90).

For left-behind children (Part B), 18 strong association rules were derived, of which 4 were 5-item sets, 12 were 4-item sets and 2 were 3-item sets. The 8 risk factors among the 12 risk factors used as LHS were included in the association rules. In addition to the two risk factors that appear in non-left-behind children, they also included low teacher support, low peer support, absence of counselors or counseling rooms in the school, low autonomy support and absence of psychology courses. The factors of female and middle school students also appeared in association rules. The high family conflict-low family cohesion, and low peer support combination had the highest correlation with depression (lift = 2.72).

Support of association rules by left-behind status ranged from 5.06% to 9.31%, confidence ranged from 80.65% to 90.74%, and lift ranged from 2.42 to 2.90.

### 3.2. Protective Factors for Nondepression: The Association Rules by Gender, Age Group and Left-Behind Status

RHS was set as the nondepression to establish association rules. By setting a minimum lift value of (X⇒Y) > 1 as the basic requirement, we compared changes in association rules and ultimately used 20% and 80% as the minimum support and confidence thresholds respectively. The combination of protective factors that meet the above parameter conditions is identified as the significant association rule in this study. The generated association rules were sorted in descending order based on their confidence levels, as presented in Table 6, Table 7 and Table 8. In tables, support reflects the probability of both protective factors and nondepression occurring simultaneously, and confidence reflects the probability of nondepression occurring under the conditions of protective factors occurring. Lift reflects the correlation between protective factors and nondepression, with a value greater than 1, indicating a positive correlation between the two.

#### 3.2.1. Protective Factors for Nondepression by Gender

Table 6 summarizes the association rules for protective factors of nondepression by gender.

For males (Part A), 12 strong association rules were derived, of which 1 was 4-item sets, 6 were 3-item sets and 5 were 2-item sets. The 7 protective factors among the 12 protective factors (shown in Table 1) used as LHS that were included in the association rules are high family cohesion, low family conflict, high peer support, high teacher support, high autonomy support, no family economic strain and no family structure risk. The factor of non-left-behind children also appeared in association rules. High peer support had the highest correlation with nondepression (lift = 1.19).

For females (Part B), 9 strong association rules were derived, of which 5 were 3-item sets and 4 were 2-item sets. The 6 protective factors among the 12 protective factors used as LHS were included in the association rules. Compared to males, the protective factors for females lack high autonomy support. High family cohesion-no family structure risk combination had the highest correlation with nondepression (lift = 1.32).

Support of association rules by gender ranged from 20.23% to 32.73%, confidence ranged from 80.32% to 88.67%, and lift ranged from 1.12 to 1.19.

#### 3.2.2. Protective Factors for Nondepression by Age Group

The association rules for protective factors of nondepression by age group are displayed in Table 7. For primary school students (Part A), 10 strong association rules were derived, of which one was 4-item sets, 4 were 3-item sets and 5 were 2-item sets. The 7 protective factors among the 12 protective factors used as LHS that are included in the association rules are high family cohesion, low family conflicts, high teacher support, high peer support, no family structure risk no family economic strain and high autonomy support. The factor of non-left-behind children also appears in association rules. High family cohesion has the highest correlation with nondepression (lift = 1.22).

For middle school students, 12 strong association rules were generated, of which 8 were 3-item sets and 4 were 3-item sets. The 7 protective factors among the 12 protective factors used as LHS were included in the association rules, which were the same as protective factors for primary school students. The factor of non-left-behind children also appeared in association rules. High family cohesion-no family structure risk combination had the highest correlation with nondepression (lift = 1.28).

Support of association rules by age group ranged from 20.03% to 33.19%, confi-dence ranged from 80.47% to 88.32%, and lift ranged from 1.19 to 1.28.

#### 3.2.3. Protective Factors for Nondepression by Left-Behind Status Group

The association rules for protective factors of nondepression by left-behind status are displayed in Table 8.

For non-left-behind children (Part A), 17 strong association rules were derived, of which 2 were 4-item sets, 10 were 3-item sets and 5 were 2-item sets. The 7 protective factors among the 12 protective factors used as LHS that were included in the association rules were high family cohesion, low family conflicts, high peer support, high teacher support, high autonomy support and without family structure risk and family economic strain. High family cohesion had the highest correlation with nondepression (lift = 1.25).

For left-behind children (Part B), 2 strong association rules were generated, both of which were 2-item sets. The only 2 protective factors among the 12 protective factors used as LHS were included in the association rules. 

Compared to non-left-behind children, the protective factors for left-behind children lack high family cohesion, high peer support, high autonomy support and without family structure risk and family economic strain. Low family conflict had the highest correlation with nondepression (lift = 1.24).

Support of association rules ranged from 20.27% to 32.53%, confidence ranged from 80.08% to 89.29%, and lift ranged from 1.14 to 1.25.

## 4. Discussion

The present study employed association rule mining to investigate the combined effects of multiple risk and protective factors of depression in family and school settings. Based on the predefined thresholds of support, confidence and lift, the strongest association rules were the combination of more than one risk/protective factor. This result supported the cumulative model of risk/protective factors that exposure to multiple risks increases the likelihood of negative outcomes while exposure to multiple protective factors predicts positive outcomes more effectively [29,34]. We found common risk/protective factors regardless of gender, age, and left-behind status and specific factors for adolescents differing in gender, age group and left-behind status.

### 4.1. Common Risk Factors for Depression and Protective Factors for Nondepression

The results indicated that risk/protective factors for depression exhibited similarities in adolescents varying in gender, age group and left-behind status. Low family cohesion and high family conflict emerged as the most influential predictive risk factors among family factors, irrespective of gender, age group and left-behind status. This result is in line with previous research highlighting the strong association between familial relational issues and depression in young people [11]. Family conflict might expose children and adolescents to violence, which potentially compromises their emotional security [36,37] and demonstrates negative emotion regulation strategies [38]. Conversely, a cohesive family environment offers supportiveness and helpfulness that contribute to the development of self-esteem in children while being closely linked to mental health outcomes such as depression [10]. In contrast, it is challenging for families lacking cohesion to provide effective social support to adolescents which consequently increases their vulnerability to depression [39].

Among school factors, low teacher support and low peer support were the most robust predictive risk factors for adolescents, irrespective of their gender, age group, or left-behind status. The findings align with previous research highlighting the pivotal role of peer and teacher support in assisting students in coping with daily stressors, fostering a sense of belongingness to school, and ultimately promoting positive mental health outcomes [40,41].

In terms of the magnitude of lift, the combinations of familial relational risk factors (high family conflict and low family cohesion) and school relational risk factors (low peer support and low teacher support) exhibited the highest predictive effect on depression, followed by the combinations of the family/school relational risks along with distal risk factors (e.g., being female, low parental education level, and absence of psychoeducational resources in the school). Factors such as low parental education level, low family socioeconomic status and female gender were not independently associated with elevated depression but rather augmented the detrimental effects of familial and school-related relational risk factors. Similar results have also been found in previous studies. For example, Westhoven (2002) found that children from families with low SES had an increased risk for internalizing symptoms, particularly in families exhibiting high levels of family conflict [42]. He et al. (2019) found that the association between interpersonal conflicts within family and school domains and depressive symptoms was stronger for girls [43]. Furthermore, we also found that a lack of psychoeducational resources in schools, especially more targeted individual counseling services, may also exacerbate the negative impact of familial or school-related relationship risks. These results suggest that schools should not only provide mental health education to all students but also identify and offer more tailored group and individual counseling services to students at risk.

As revealed by our results, high family cohesion, low family conflict, high teacher support and high peer support are consistent protective factors against depression among adolescents across different genders, age groups and left-behind statuses. Notably, the effects of these protective factors were amplified when they were accompanied by additional factors such as the absence of family structure risk, the absence of family economic strain, and living with parents. This result is consistent with the protection-protective factor hypothesis, which suggests that the effect of a protective factor increases with the presence of other protective factors [44].

### 4.2. Gender Differences in Risk Factors for Depression and Protective Factors for Nondepression

In line with the gender difference in depression prevalence, we found far more strong association rules for depression in girls compared to those in boys. This result is consistent with the notion that adolescent girls might encounter more stressful life events than boys due to gender inequality and heightened social pressure on gender roles in adolescence. In addition, the hyperactivity of the hypothalamic-pituitary-adrenal axis (HPA) and a greater tendency towards rumination in females might also contribute to their increased vulnerability to depression during adolescence [23].

In addition, we found that low teacher support was more related to depression in boys while low peer support was more associated with depression in girls. It is in line with the social orientation of self-concept in females [23]. Girls display heightened sensitivity towards peer relations problems, which consequently increases their susceptibility to experiencing negative emotions.

### 4.3. Age Group Differences in Risk Factors for Depression and Protective Factors for Nondepression

For primary school students, only the combination of low family cohesion and low peer support was strongly associated with depression, whereas there were 30 strong association rules for risk factors for middle school students. This result aligns with previous research suggesting that middle school students are more susceptible to depression compared to their primary school counterparts [25], emphasizing the imperative need for implementing school-based psychoeducation and psychological services for middle school students.

### 4.4. Risk Factors for Depression and Protective Factors for Nondepression by Left-Behind Status

Regarding the association rules for risk factors of depression by left-behind status, we obtained stronger association rules for left-behind children compared to those of non-left-behind children. Low family cohesion and high family conflict were still the strongest predictors of depression. The lifts were higher when female gender, middle school students and other distal factors were combined. Peer support and teacher support can also significantly predict depression in left-behind children, especially when combined with family relational risk factors.

In terms of protective factors, the protective factors of left-behind children were significantly fewer than those of non-left-behind children. Low family conflict was still an important protective predictor of left-behind children. In addition, teacher support was also closely related to depression. For left-behind children, due to insufficient family support resources, the role of school resources is more important. Especially, teachers can help left-behind adolescents better solve difficulties and problems encountered in study and life, which is especially important to protect their mental health [45].

## 5. Conclusions

In this study, according to different age, gender and left-behind status groups, the combinations of risk/protective factors that affect depression were examined by utilizing association rules analysis. The analysis results reveal strong associations between familial relational factors and school-related relational factors, with adolescent depression. When these factors are combined, the risk of depression in adolescents increases. Moreover, female gender, middle school students, low family socioeconomic status, family structural risk, separation from parents, and a lack of mental health education resources at school exacerbate the negative impact of the aforementioned risk factors. Furthermore, the risk and protective factors for depression varied according to gender, age group and left-behind status.

### 5.1. Implications

The results of this study have some implications for the prevention and control of depression among adolescents. First, it is crucial to identify the population at risk as a fundamental prerequisite for effective intervention. Our research revealed that common risk factors include high family conflict, low family cohesion, low peer support, and low teacher support. When these factors are combined, the likelihood of depression in adolescents increases substantially. The risk of depression further improves when combined with other factors such as being female, being middle school students, separating from parents, having a low family socioeconomic status and having low family economic difficulties. These vulnerable teenagers require heightened attention and targeted support. Secondly, informed by the results, it is imperative to prioritize teacher-student relationships and peer relationships as important targets in school-based mental health education programs, aiming to create a harmonious and friendly campus atmosphere. At the same time, parents’ education should be included to cultivate a warm and positive family atmosphere for the positive development of adolescents. Furthermore, the focus of mental health education should be clarified according to the differences in gender, age, and left-behind status. Given that girls are more susceptible to interpersonal stress compared to boys, greater emphasis may be placed on addressing peer relationship issues for them. For left-behind children with limited familial resources, particular attention should be given to fostering a supportive campus atmosphere and nurturing healthy teacher-peer relations. Additionally, apart from general mental health education, it is important to provide school-based mental health services such as group counseling sessions and individual therapy to adolescents at risk.

### 5.2. Limitations and Future Direction

This study has certain limitations. Firstly, the inclusion of school and family environmental factors in this study remains limited, making it challenging to comprehensively reflect the risk and protective factors across different groups. Future research should consider additional environmental factors associated with depression, such as parent-child relationships and academic stress. Secondly, this study solely focuses on external environmental risk and protective factors for adolescent depression. However, individual differences may interact with these environmental factors and influence psychological adaptation. Therefore, future studies should incorporate individual differences into analyses. Despite these limitations, the results of this study suggested that applying association rules to a large sample of student mental health research data yields meaningful results. If additional data is accumulated or the survey scope is expanded in the future, applying this analysis method is not only possible but also has the potential to generate more accurate and reliable psychological risk screening standards.

## Figures and Tables

**Table 1:** Predictors included in this study with criterion of risk and protection ^1^.

Category	Description	Criteria for Determining Risk Factors	Criteria for Determining Protective Factors
Familial factors			
Family structure	Parental marital status and whether the child lives with both parents	Not living with both parents because of parental divorce or separation	living with both parents
Separation from parents (left-behind status)	Whether the child separates from parents for a long time because parents go out for work	Have not lived with at least one of the parents for 6 months because their parents have been out at work	No separation from any parents in the last 6 months
Parental education	Highest grade completed by the parents	Parental education level was below junior high school	Parental education level was junior high school and above
Financial strain	How often has the family faced specific economic problems in the past year	≥75th percentile of the total score	≤25th percentile of the total score
Family cohesion	Closeness among family members	≤25th percentile of the total score	≥75th percentile of the total score
Family conflict	Frequencies of conflicts among family members	≥75th percentile of the total score	≤25th percentile of the total score
School-related factors			
Psychological courses	provision of mental health education in schools	Absence of psychological courses	Offering regular psychological courses
Psychological counsellors	provision of mental health services in schools	Absence of psychological counselors	Psychological counselors are available in the school
Psychological counseling rooms	provision of mental health facilities in schools	Absence of psychological counseling rooms	Equipped with psychological counseling rooms in the school
Teacher support	Teachers’ instrumental and emotional support	≤25th percentile of the total score	≤25th percentile of the total score
Peer support	Peer relationship in the school	≤25th percentile of the total score	≤25th percentile of the total score
Autonomy support	opportunities provided for autonomy in the school	≤25th percentile of the total score	≤25th percentile of the total score

^1^ Academic ranking was also collected and used as predictors in analyses. Students were asked to report whether they were usually the first 10 in the class, at the average or the last 10 in the class. Due to insufficient evidence regarding the criteria for determining the risk or protective factors in academic ranking, we refrained from defining specific criteria but included this variable in our analyses for exploratory purposes.

**Table 2:** Definition of formula and explanation of support, confidence and lift.

Parameter	Formula (P Is the Probability of an Association)	Meaning
Support	$\sup\left(X\Rightarrow Y\right)=P\left(X\cap Y\right)$	Support refers to the probability of {X, Y} appearing in all item sets, that is the probability that the item set contains both X and Y.
Confidence	$\mathrm{conf}\left(X\Rightarrow Y\right)=P\left(Y|X\right)$	Confidence represents the probability of occurrence of the RHS Y under the condition that the LHS X occurs, that is, the probability of containing Y in the item set that contains X.
Lift	$\mathrm{lift}\left(X\Rightarrow Y\right)=P\left(X\cap Y\right)/P\left(X\right)P\left(Y\right)$	The lift measures the dependency between X and Y. Lift values near 1 indicate X and Y almost often appear together as expected, greater than 1 means they appear together more than expected and less than 1 means they appear less than expected. Greater lift values indicate a stronger association.

**Table 3:** Association rules for risk factors of depression by gender.

LHS (Both of RHS Are Depression)	Support	Confidence	Lift
Part A: Male			
low family cohesion, high family conflict	5.56%	78.05%	3.04
low family cohesion, low teacher support	5.30%	70.93%	2.76
Part B: Female			
low family cohesion, high family conflict, absence of counselors in the school	5.03%	89.04%	2.61
high family conflict, low peer support	6.27%	88.04%	2.59
low family cohesion, high family conflict, middle school students	6.11%	86.81%	2.55
low family cohesion, high family conflict	8.13%	86.78%	2.55
low family cohesion, low peer support	7.97%	85.12%	2.50
low family cohesion, low peer support, absence of counselors in the school	5.26%	85.00%	2.50
low family cohesion, middle school students, absence of counseling rooms in the school	5.80%	79.79%	2.34
low peer support, low teacher support	5.57%	78.26%	2.30
low family cohesion, middle school students, absence of counselors in the school	6.11%	78.22%	2.30
low family cohesion, middle school students, absence of counselors in the school, absence of counseling rooms in the school	5.26%	78.16%	2.30
low family cohesion, middle school students	9.91%	78.05%	2.29
low family cohesion, low teacher support	5.65%	76.04%	2.23
high family conflict, middle school students, absence of counselors in the school	5.80%	75.76%	2.22
high family conflict, separation from parents	5.26%	74.73%	2.19
low peer support, middle school students	8.44%	74.66%	2.19
high family conflict, middle school students, absence of counseling rooms in the school	5.42%	74.47%	2.19
low family cohesion, middle school students, low parental education level	5.73%	74.00%	2.17
high family conflict, absence of counselors in the school	7.89%	73.38%	2.15
high family conflict, absence of counselors in the school, absence of counseling rooms in the school	6.81%	73.33%	2.15
high family conflict, absence of counseling rooms in the school	7.43%	72.73%	2.14
low peer support, absence of counselors in the school	9.29%	72.29%	2.12
low peer support, low parental education level, absence of counselors in the school	5.03%	72.22%	2.12
low peer support, average academic ranking	6.81%	72.13%	2.12
low peer support, middle school students, low parental education level	5.11%	71.74%	2.11
low peer support, separation from parents	5.65%	70.87%	2.08
high family conflict, average academic ranking	5.57%	70.59%	2.07
low peer support, family economic strain	5.19%	70.53%	2.07
high family conflict, absence of counselors in the school, absence of psychological courses	5.11%	70.21%	2.06

**Table 4:** Association rules for risk factors of depression by age group.

LHS (Both of RHS Are Depression)	Support	Confidence	Lift
Part A: Primary school students			
low family cohesion, low peer support	7.39%	70.49%	2.54
Part B: Middle school students			
high family conflict, low family cohesion, low peer support	5.23%	90.54%	2.81
high family conflict, low family cohesion, female	6.17%	86.81%	2.69
high family conflict, low family cohesion, low parental education level	5.31%	86.08%	2.67
high family conflict, low family cohesion, absence of counselors in the school	5.62%	85.71%	2.66
high family conflict, low family cohesion, absence of counseling rooms in the school	5.39%	85.19%	2.64
high family conflict, low peer support	6.48%	84.69%	2.63
high family conflict, low family cohesion	9.06%	84.67%	2.63
low peer support, low family cohesion	7.88%	80.80%	2.51
low peer support, low family cohesion, absence of counselors in the school	5.15%	80.49%	2.50
low family cohesion, absence of counseling rooms in the school, female	5.85%	79.79%	2.47
low family cohesion, absence of counselors in the school, female	6.17%	78.22%	2.43
low family cohesion, female, absence of counseling rooms in the school, absence of counselors in the school	5.31%	78.16%	2.42
low family cohesion, female	9.99%	78.05%	2.42
low family cohesion, low teacher support	5.70%	77.66%	2.41
high family conflict, female, absence of counselors in the school	5.85%	75.76%	2.35
low family cohesion, family economic strain	5.62%	75.00%	2.33
low peer support, female	8.51%	74.66%	2.32
high family conflict, female, absence of counseling rooms in the school	5.46%	74.47%	2.31
low family cohesion, female, Low parental education level	5.78%	74.00%	2.30
low family cohesion, low autonomy support	6.01%	72.64%	2.25
low family cohesion, absence of counselors in the school	9.76%	71.84%	2.23
low peer support, female, low parental education level	5.15%	71.74%	2.23
high family conflict, absence of counselors in the school	7.88%	71.63%	2.22
low family cohesion, absence of counseling rooms in the school	9.45%	71.60%	2.22
high family conflict, separation from parents	5.70%	71.57%	2.22
high family conflict, absence of counselors in the school, absence of counseling rooms in the school	6.79%	71.31%	2.21
low family cohesion, absence of counseling rooms in the school, absence of psychological counselors	8.51%	71.24%	2.21
low family cohesion, absence of psychology courses	8.27%	70.67%	2.19
low family cohesion, absence of counselors in the school, absence of psychology courses	7.03%	70.31%	2.18
high family conflict, absence of counseling rooms in the school	7.73%	70.21%	2.18

**Table 5:** Association rules for risk factors of depression by left-behind status group.

LHS (Both of RHS Are Depression)	Support	Confidence	Lift
Part A: Non-left-behind children			
high family conflict, low family cohesion	5.59%	82.24%	2.90
Part B: left-behind children			
high family conflict, low family cohesion, low peer support	5.63%	90.74%	2.72
low family cohesion, high family conflict, absence of counselors in the school, absence of counseling rooms in the school	6.32%	87.30%	2.62
low family cohesion, high family conflict, absence of counseling rooms in the school	6.44%	86.15%	2.58
low family cohesion, high family conflict, female	5.63%	85.96%	2.58
low family cohesion, high family conflict, middle school students	6.21%	85.71%	2.57
low family cohesion, high family conflict, absence of counselors in the school	7.47%	85.53%	2.57
low family cohesion, high family conflict, absence of psychology courses, absence of counselors in the school	5.17%	84.91%	2.55
low family cohesion, low peer support, female	5.63%	84.48%	2.53
low family cohesion, high family conflict, absence of psychology courses, absence of counselors in the school	5.63%	84.48%	2.53
low family cohesion, high family conflict	9.31%	84.38%	2.53
low family cohesion, low teacher support, low peer support	5.98%	83.87%	2.52
low family cohesion, low teacher support, low peer support, absence of counselors in the school	5.06%	83.02%	2.49
high family conflict, low peer support	6.90%	82.19%	2.47
low family cohesion, low teacher support, absence of psychology courses, absence of counselors in the school	5.29%	82.14%	2.46
low family cohesion, low teacher support, absence of psychology courses	5.75%	81.97%	2.46
low family cohesion, low peer support, middle school students	5.17%	81.82%	2.45
low family cohesion, low teacher support, low autonomy support	5.63%	81.67%	2.45
high family conflict low peer support, absence of counselors in the school	5.75%	80.65%	2.42

**Table 6:** Association rules for protective factors of nondepression by gender.

LHS (Both of RHS Are Nondepression)	Support	Confidence	Lift
Part A: Male			
high peer support	23.09%	88.67%	1.19
high family cohesion	21.61%	87.99%	1.18
low family conflict, no family economic strain	25.00%	87.01%	1.17
low family conflict, no family structure risk, no family economic strain	20.75%	86.91%	1.17
low family conflict, no family structure risk	26.91%	86.35%	1.16
low family conflict, non-left-behind children	22.14%	86.15%	1.16
low family conflict	32.73%	85.88%	1.16
high teacher support, no family structure risk	22.74%	85.34%	1.15
high autonomy support, no family structure risk	20.23%	85.04%	1.14
high teacher support	28.30%	84.24%	1.13
high teacher support, no family economic strain	20.31%	83.27%	1.12
high autonomy support	25.35%	82.95%	1.12
Part B: Female			
high family cohesion, no family structure risk	20.43%	87.13%	1.32
high family cohesion, no family economic strain	20.82%	86.50%	1.31
high family cohesion	23.99%	86.35%	1.31
high peer support, no family structure risk	20.74%	84.54%	1.28
high peer support, no family economic strain	21.67%	83.83%	1.27
high peer support	25.62%	82.96%	1.26
low family conflict, no family structure risk	23.07%	82.09%	1.24
low family conflict	28.48%	80.35%	1.22
high teacher support	23.07%	80.32%	1.22

**Table 7:** Association rules for protective factors of nondepression by age group.

LHS (Both of RHS Are Nondepression)	Support	Confidence	Lift
Part A: Primary school students			
high family cohesion	20.81%	88.32%	1.22
high teacher support, no family structure risk	21.15%	88.17%	1.22
high peer support	24.51%	86.63%	1.20
low family conflict, no family structure risk	26.31%	85.24%	1.18
low family conflict, no family structure risk, no family economic strain	20.03%	84.73%	1.17
high teacher support	27.09%	84.22%	1.17
low family conflict	33.19%	83.55%	1.16
low family conflict, not left-behind children	21.58%	83.11%	1.15
low family conflict, no family economic strain	25.37%	83.10%	1.15
high autonomy support	22.36%	82.02%	1.14
Part B: Middle school students			
high family cohesion, no family structure risk	21.55%	86.79%	1.28
high family cohesion	24.75%	86.14%	1.27
high peer support, no family structure risk	20.69%	86.04%	1.27
high family cohesion, no family economic strain	20.37%	85.29%	1.26
high peer support	24.36%	84.32%	1.24
high peer support, no family economic strain	20.45%	83.97%	1.24
not left-behind children, low family conflicts	20.37%	83.39%	1.23
low family conflict, no family economic strain	23.03%	83.33%	1.23
low family conflict, no family structure risk,	23.58%	83.20%	1.23
low family conflict	28.02%	82.53%	1.22
high autonomy support, no family structure risk	21.86%	80.69%	1.19
high teacher support	24.12%	80.47%	1.19

**Table 8:** Association rules for protective factors of nondepression by left-behind status.

LHS (Both of RHS Are Nondepression)	Support	Confidence	Lift
Part A: non-left-behind children			
high family cohesion	26.49%	89.29%	1.25
high family cohesion, no family structure risk	23.82%	89.29%	1.25
high family cohesion, no family economic strain, no family structure risk	20.27%	88.37%	1.23
high family cohesion, no family economic strain	22.49%	88.28%	1.23
high peer support, no family structure risk	23.70%	87.35%	1.22
high peer support, no family economic strain	22.62%	86.20%	1.20
high peer support	26.94%	86.00%	1.20
high teacher support, no family structure risk	23.57%	84.13%	1.17
high teacher support, no family economic strain	20.97%	83.76%	1.17
low family conflict, no family economic strain, no family structure risk	23.25%	83.56%	1.17
low family conflict, no family structure risk	28.34%	83.52%	1.17
high teacher support	26.37%	83.50%	1.17
low family conflict	32.53%	83.25%	1.16
low family conflict, no family economic strain	26.49%	83.23%	1.16
high autonomy support, no family economic strain	20.46%	83.20%	1.16
high autonomy support, no family structure risk	22.17%	83.10%	1.16
high autonomy support	25.35%	81.43%	1.14
Part B: left-behind children			
low family conflict	26.78%	82.62%	1.24
high teacher support	24.02%	80.08%	1.20

## Data Availability

Data is available upon reasonable request.

## References

[1] Thapar A., Eyre O., Patel V., Brent D. (2022). Depression in young people. Lancet.

[2] Li F., Cui Y., Li Y., Guo L., Ke X., Liu J., Luo X., Zheng Y., Leckman J.F. (2022). Prevalence of mental disorders in school children and adolescents in China: Diagnostic data from detailed clinical assessments of 17,524 individuals. J. Child Psychol. Psychiatry.

[3] Fu X., Zhang K., Chen X., Chen Z. (2023). Report on National Mental Health Development in China (2021–2022).

[4] Bronfenbrenner U. (1979). The Ecology of Human Development: Experiments by Nature and Design.

[5] Zhou Q., Fan L., Yin Z. (2018). Association between family socioeconomic status and depressive symptoms among Chinese adolescents: Evidence from a national household survey. Psychiatry Res..

[6] Ruttle P.L., Armstrong J.M., Klein M.H., Essex M.J. (2014). Adolescent internalizing symptoms and negative life events: The sensitizing effects of earlier life stress and cortisol. Dev. Psychopathol..

[7] Chang L.-Y., Fu M. (2020). Disentangling the effects of intergenerational transmission of depression from adolescence to adulthood: The protective role of self-esteem. Eur. Child Adolesc. Psychiatry.

[8] Li N., Li Y., Huang X., Xiang S., Hu Q., Luo C., Ju P., Mellor D., Xu Y., Fei H. (2022). The role of achievement attribution in the associations between parent-child communication and psychological well-being among adolescents: A mediation analysis. Eur. Psychiatry.

[9] Tang A.-M., Deng X.-L., Du X.-X., Wang M.-Z. (2018). Harsh parenting and adolescent depression: Mediation by negative self-cognition and moderation by peer acceptance. Sch. Psychol. Int..

[10] Lin W.-H., Yi C.-C. (2019). The Effect of Family Cohesion and Life Satisfaction During Adolescence on Later Adolescent Outcomes A Prospective Study. Youth Soc..

[11] Tang X., Tang S., Ren Z., Wong D.F.K. (2020). Psychosocial risk factors associated with depressive symptoms among adolescents in secondary schools in mainland china: A systematic review and meta-analysis. J. Affect. Disord..

[12] Mackova J., Veselska Z.D., Bobakova D.F., Geckova A.M., van Dijk J.P., Reijneveld S.A. (2019). Crisis in the Family and Positive Youth Development: The Role of Family Functioning. Int. J. Environ. Res. Public Health.

[13] Richardson C., Oar E., Fardouly J., Magson N., Johnco C., Forbes M., Rapee R. (2019). The Moderating Role of Sleep in the Relationship Between Social Isolation and Internalising Problems in Early Adolescence. Child Psychiatry Hum. Dev..

[14] Ye Z., Wu D., He X., Ma Q., Peng J., Mao G., Feng L., Tong Y. (2023). Meta-analysis of the relationship between bullying and depressive symptoms in children and adolescents. BMC Psychiatry.

[15] Jiang S., Ren Q., Jiang C., Wang L. (2021). Academic stress and depression of Chinese adolescents in junior high schools: Moderated mediation model of school burnout and self-esteem. J. Affect. Disord..

[16] Guo J., Liu L., Zhao B., Wang D. (2020). Teacher Support and Mental Well-Being in Chinese Adolescents: The Mediating Role of Negative Emotions and Resilience. Front. Psychol..

[17] Zhang D., Jin B., Cui Y. (2022). Do Teacher Autonomy Support and Teacher-Student Relationships Influence Students’ Depression? A 3-Year Longitudinal Study. Sch. Ment. Health.

[18] van Loon A.W.G., Creemers H.E., Beumer W.Y., Okorn A., Vogelaar S., Saab N., Miers A.C., Westenberg P.M., Asscher J.J. (2020). Can Schools Reduce Adolescent Psychological Stress? A Multilevel Meta-Analysis of the Effectiveness of School-Based Intervention Programs. J. Youth Adolesc..

[19] Zhang Z., Wang Y., Zhao J. (2023). Longitudinal Relationships Between Interparental Conflict and Adolescent Depression: Moderating Effects of School Connectedness. Child Psychiatry Hum. Dev..

[20] Lee W.K., Joo Y.S. (2022). Examining family processes linked to adolescent problem behaviors in single-mother families: The moderating role of school connectedness. Front. Psychol..

[21] Gan X., Li H., Xiang G.-X., Lai X.-H., Jin X., Wang P.-Y., Zhu C.-S. (2022). Cumulative Family Risk and Cyberbullying Among Chinese Adolescents: The Chain Mediating Role of School Connectedness and Cyber Victimization. Front. Public Health.

[22] Salk R.H., Hyde J.S., Abramson L.Y. (2017). Gender Differences in Depression in Representative National Samples: Meta-Analyses of Diagnoses and Symptoms. Psychol. Bull..

[23] Nolen-Hoeksema S. (2001). Gender differences in depression. Curr. Dir. Psychol. Sci..

[24] Hankin B.L., Mermelstein R., Roesch L. (2007). Sex differences in adolescent depression: Stress exposure and reactivity models. Child Dev..

[25] Xu D.-D., Rao W.-W., Cao X.-L., Wen S.-Y., An F.-R., Che W.-I., Bressington D.T., Cheung T., Ungvari G.S., Xiang Y.-T. (2020). Prevalence of depressive symptoms in primary school students in China: A systematic review and meta-analysis. J. Affect. Disord..

[26] Liang Y., Wang L., Rui G. (2017). Depression among left-behind children in China. J. Health Psychol..

[27] Olson D., Portner J., Bell R. (1985). Family Adaptation and Cohesion Evaluation Scales II-Family Version (FACES-II).

[28] Fei L.P., Shen Q.J., Zheng Y.P., Zhao J.P., Jiang S.A., Wang L.W., Wang X.D. (1991). Preliminary evaluation of Chinese version of FACES II and FES: Comparison of normal families and families of schizophrenic. Chin. Ment. Health J..

[29] Evans G.W., Li D., Whipple S.S. (2013). Cumulative Risk and Child Development. Psychol. Bull..

[30] Moos R.H., Moos B.S. (1981). Manual for the Family Environment Scale.

[31] Jia Y., Way N., Ling G., Yoshikawa H., Chen X., Hughes D., Ke X., Lu Z. (2009). The influence of student perceptions of school climate on socioemotional and academic adjustment: A comparison of Chinese and American adolescents. Child Dev..

[32] Birleson P. (1981). The validity of depressive disorder in childhood and the development of a self-rating scale: A research report. J. Child Psychol. Psychiatry.

[33] Su L. (2003). Norm of the depression self-rating scale for children in Chinese urban children. Chin. Ment. Health J..

[34] Agrawal R., Imielinski T., Swami A. Mining association rules between sets of items in large databases. Proceedings of the ACM Sigmod Conference on Management of Data.

[35] Agrawal R., Srikant R. Fast algorithms for mining association rules. Proceedings of the 20th Very Large Data Bases Conference, Santiago de.

[36] Kim B.K.E., Gilman A.B., Hill K.G., Hawkins J.D. (2016). Examining protective factors against violence among high-risk youth: Findings from the Seattle Social Development Project. J. Crim. Justice.

[37] Luo R., Chen F., Ke L., Wang Y., Zhao Y., Luo Y. (2022). Interparental conflict and depressive symptoms among Chinese adolescents: A longitudinal moderated mediation model. Dev. Psychopathol..

[38] Morris A.S., Criss M.M., Silk J.S., Houltberg B.J. (2017). The Impact of Parenting on Emotion Regulation During Childhood and Adolescence. Child Dev. Perspect..

[39] Fredrick S.S., Nickerson A.B., Livingston J.A. (2022). Family cohesion and the relations among peer victimization and depression: A random intercepts cross-lagged model. Dev. Psychopathol..

[40] Gao J.-L., Wang L.-H., Yin X.-Q., Hsieh H.-F., Rost D.H., Zimmerman M.A., Wang J.-L. (2021). The promotive effects of peer support and active coping in relation to negative life events and depression in Chinese adolescents at boarding schools. Curr. Psychol..

[41] Letkiewicz A.M., Li L.Y., Hoffman L.M.K., Shankman S.A. (2023). A prospective study of the relative contribution of adolescent peer support quantity and quality to depressive symptoms. J. Child Psychol Psychiatry.

[42] Westhoven V.C. (2002). The Longitudinal Effect of Perceived and Observed Family Functioning on Internalizing Symptoms in Young Adolescents with Spina Bifida. Ph.D. Thesis.

[43] He G.-H., Strodl E., Chen W.-Q., Liu F., Hayixibayi A., Hou X.-Y. (2019). Interpersonal Conflict, School Connectedness and Depressive Symptoms in Chinese Adolescents: Moderation Effect of Gender and Grade Level. Int. J. Environ. Res. Public Health.

[44] Christiansen E.J., Evans W.P. (2005). Adolescent victimization—Testing models of resiliency by gender. J. Early Adolesc..

[45] Huang L., Zhang S., Bian B., Zhou M., Bi Z. (2023). Peer effects of depression between left-behind and non-left-behind children: Quasi-experimental evidence from rural China. Child Adolesc. Psychiatry Ment. Health.

